# Exploring time and frequency linkages of green bond with renewable energy and crypto market

**DOI:** 10.1007/s10479-022-05074-8

**Published:** 2022-12-02

**Authors:** Miklesh Prasad Yadav, Priyanka Tandon, Anurag Bhadur Singh, Adam Shore, Pali Gaur

**Affiliations:** 1 Indian Institute of Foreign Trade, Kakinada, India; 2 Regenesys Business School, Sandton, South Africa; 3 School of Commerce, XIM University, Bhubaneswar, Odisha India; 4 Liverpool Business School, Liverpool John Moores University, Redmonds Building, Brownlow Hill Liverpool, Merseyside, L3 5UG UK

**Keywords:** Dynamic linkages, Green bond, Renewable energy, Cryptocurrency

## Abstract

This paper examines the dynamic linkages of green bond with the energy and crypto market. The S&P green bond index (RSPGB) is used as a proxy for the green bond market; S&P global clean energy index and ISE global wind energy (RIGW) are used as proxies for the renewable energy market, and; Bitcoin and Ethereum (RETHER) are used as the proxies of the crypto market. The daily prices of these constituent series are collected using Bloomberg from October 3, 2016 to February 23, 2021. We undertake an empirical analysis through the application of three key tests, namely: dynamic conditional correlation (DCC), Diebold and Yilmaz (Int J Forecast 28(1):57–66, 2012. 10.1016/j.ijforecast.2011.02.006), Baruník and Křehlík (J Financ Econom 16(2):271–296, 2018. 10.1093/jjfinec/nby001) model. The DCC reveals no dynamic linkages of volatility from the green bond to the energy and crypto market in the short run. Referring to Diebold and Yilmaz (2012), it dictates that the green bond (RSPGB) is a net receiver while the energy market (RIGW) and cryptocurrency (RETHER) are the largest and least contributors to the transmission of the volatility. Additionally, the Baruník and Křehlík (2018) model confirmed that the magnitude of the total spillover is high in more prolonged than shorter periods, suggesting reduced diversification opportunities. Overall, the present study exemplifies the significance of the green bond market as protection against risk.

## Introduction

Economic development for any nation has pros and cons reflected by technological upgradation and environmental degradation, where in some instances the former also contributes to the negative climate implications. Sustainable economic development is acknowledged as a solution to this degradation (UNESCO, 2011), which can be achieved by ‘greening’ the economy with the help of green bonds (Jin et al., 2020). Green bonds are structured instruments, similar to traditional fixed income corporate bonds, except that proceeds are designated for environmentally friendly projects (Reboredo & Ugolini, 2020) and are considered ideal for diversification in the portfolios of environmentally concerned investors.

Additionally, renewable energy as an alternative to fossil fuels also supplements the carbon emissions reduction to achieve a sustainable economy as mentioned in Paris Agreement 2015 and UN Sustainable Development Goals (Kumar et al., 2012a; b). Kyritsis and Serletis (2019) argued that crude oil is a leading energy source for power generation and hence examined the probable effect of its price uncertainties on renewable energy consumption. Interestingly, involvement in crypto validation requires a high amount of power consumption which converts into significant carbon emissions (Stoll et al., 2019). Hence, spillover between energy market and crypto market is of major concern.

The economic development phase of any economy can be segregated as pre-industrialization phase (scale effect), industrialization phase (composition effect), and post industrialization phase (technique effect) (Dogan & Inglesi-lotz, 2020; Balsalobre-Lorente et al. 2019; Shahbaz et al. 2019). Being in post-Industrialization phase, the use of cryptocurrency has become a lucrative investment avenue for investors and they serve as a better diversification option (Gil-Alana et al. 2020). Additionally, there has been much discussion regarding the potential impact of bitcoin and other cryptocurrencies on financial markets and transactions but the repercussions of bitcoin demand on the environment, however, have gone unnoticed. The technological progress reflecting the economic development also comes at the cost of environmental dilapidation. The advancement of digitization and use of cryptocurrencies, for example, has resulted in increased demand for energy in the form of electricity, the high consumption of which adds significantly to the global carbon emissions. The meteoric rise of Bitcoin and other cryptocurrencies has captivated the public's attention in recent years (Akyildirim et al., 2021). The global cryptocurrency market size in 2020 is valued at $1.49 billion, reaching $4.94 billion by 2030. Cryptocurrencies like Bitcoin and Ethereum have gained massive popularity in the last decade due to high returns and use in portfolio diversification (Yan et al., 2022). On the one hand, this thriving new technology is gaining traction in our global economy because it fosters trans-business and investment activity. At the same time, regulators are concerned that cryptocurrencies could sponsor illicit activity (Koutmos, 2020). Another concerning issue relating to cryptocurrencies, especially bitcoin, is; that Bitcoin is a turbulent asset, with most transactions aimed at speculative investments (Cretarola & Figa-Talamanca, 2021). However, investors are being compensated with premiums for their risky investments in Bitcoin (Bouri et al., 2022). There have been numerous studies conducted investigating the volatility of Bitcoin during the pandemic (Ftiti et al., 2021). Though, looking at the environmental aspect, electricity consumption while crypto trading and the undertaking of calculations have been a turning point in the case of cryptocurrencies. This is more prevalent in countries with high coal consumption for mining like China as the top contender, as estimated by The Cambridge Bitcoin Electricity Consumption Index (CBECI), adding to the carbon emissions in a significant way with many other nations like Denmark and Norway (Yan et al., 2022). Regardless of other environmental penalties, Bitcoin and other cryptocurrencies could drive global warming above 2 °C and be considered power-hungry currencies due to high electricity consumption (Mora et al., 2018). Besides, this market is impacted significantly by renewable energy stocks and carbon pricing to reduce the use of conventional energy sources. Sustainable economic development and climate change have caught the attention of global leaders for fighting the overall environmental degradation. The United Nations 2018 included clean climate in their Sustainable Development Goals (SDG) to fight all environmental concerns. International agreements like the Paris Agreement in 2015 and European Green Deal in 2019 have contributed significantly to achieving the given targets of greening the economy (Naeem et al., 2021a, 2021b), however, a substantial amount of investment is needed to unravel the issue of financing the greening process. The adoption of green bonds has emerged as a new phenomenon in the global economy's healing process by developing low-carbon technological innovations to reduce carbon emissions (Laskowska, 2018; Monk & Perkins, 2020). In the case of renewable energy projects or environmental evolutions like water projects, green bonds have come to the forefront of the realignment toward a climate-resilient economy (Tolliver et al., 2020). The same has been considered as output or value-added term which is integral part of operation management. In this paper, the output is linking pin to the diversification opportunities which can be feasible in investigating the connectedness among constituent markets (Green bond, Renewable energy and Crypto-market). It has become more and more strategic in driving all the financial aspects due to which this paper covers the need of operation research. Generally, value of output in operation management is measured by the prices that stakeholders (customers) pay for goods and services and many factors affect these outputs. In consideration of these issues, this paper answers the following research questions:

1. Is green bond connected with renewable energy and the crypto-market in various time frequencies such as short, medium and long?
2. Is the output (diversification) accentuated from the input in the form of connectedness amongst green bonds, renewable energy and crypto market?

According to Markowitz's Modern Portfolio Theory by Markowitz (1952, 1991), investors should make sure to diversify their security holdings by investing in a variety of economically varied industries. Other theories that are connected to diversification investment strategies include the behavioural finance theory (BFT), efficient market hypothesis (EMH), and capital asset pricing model (CAPM) (Letho et al., 2022). The CAPM suggests that an investor chooses a higher future value of an investment over a lower future value (Sharpe, 1964). From a psychological and sociological standpoint, the BFT evaluates the financial markets and investors (Malkiel, 2015; Subrahmanyam, 2007). Hence, it is imperative to investigate the interconnectedness of various financial markets and crypto market for providing the valuable insights to investors keeping in mind the sustainability as important factor. Going through the extant literature, it can be inferred that sustainable energy stocks and the use of green bonds for diversifying the investor’s asset allocation have been emphasized in many studies focussing primarily on volatility studies. However, dynamic linkages of green bonds and renewable energy with cryptocurrencies and their impact on the environment are not robustly studied. Likewise, because the mechanisms of return and volatility linkages may diverge, both may provide value—relevant information to investors. Furthermore, the connectivity may vary over time, which must be estimated using an appropriate model. An optimal decision making with financial modelling and risk management techniques has been playing a key role in the analysis and understanding of financial market dynamics (Board et al., 2003) through operation research, while the tools and techniques of predictive modelling and quantitative forecasting from operations research/management are used for real time analysis. Hence, the present study utilises different econometric models for quantitative forecasting (Fildes, 1985) of the time and frequency linkages of green bond, with renewable energy and crypto market. As a result, this study will use a Diebold and Yilmaz (2012) and Baruník and Křehlík (2018) model which is an optimal estimator of time-variation in parameters, to analyze the time-varying connectedness of Bitcoin, Etherium, clean energy, and green bond prices in return and volatility. Although, International agreements like the Paris Agreement in 2015 and European Green Deal in 2019 have contributed significantly in achieving the given targets of greening the economy (Naeem et al., 2021a, 2021b). However, a substantial amount of investment is needed to unravel the issue of financing the greening process.

With this backdrop, the current study attempts to unravel the dynamic linkages between green bond, clean energy market and the crypto market. Here, S&P green bond index (RSPGB), S&P global clean energy index (RSPCE), ISE global wind energy (RIGW), Bitcoin (RBIT), and Ethereum (RETHER) represent the green bond, renewable energy, and crypto-market respectively. The reason behind choosing Bitcoin is that it has become one of the most traded assets in recent years, piquing interest in both industry and academia (Deng et al., 2021). On a similar note, Cai et al. (2021) found the effect of price explosivity on the returns of Bitcoin. We apply dynamic conditional correlation (DCC), Diebold and Yilmaz (2012), Baruník and Křehlík (2018) model based on daily data. We inferred that there are no dynamic linkages of volatility from the green bond to the energy and crypto market in the short run. Further, Diebold and Yilmaz (2012) reveal that the green bond (RSPGB) is a net receiver while the energy market (RIGW) and cryptocurrency (RETHER) are the largest and least contributors to the transmission of the volatility. Finally, application of Baruník and Křehlík's (2018) model demonstrates that the magnitude of the total spillover is higher in the long run than in the short run. The study's findings align with (Chai et al., 2022) who confirmed the interconnectedness of green bonds and clean energy stocks. This paper contributes to existing studies in fourfold: First, we demonstrated statistically that the energy market (RIGW) and cryptocurrency (RETHER) are the largest and smallest contributors to the volatility transmission respectively. Second, unlike earlier research, we have quantified the dynamic and time-varying connectivity between distinct clean energy indexes, green bonds, and cryptocurrencies. Third, the study discovered that, total connectedness amongst five series (green bonds, clean energy, wind energy, Bitcoin and Ethereum) is observed to be higher in the long-run than in the short run, thus suggesting reduced diversification opportunities for the investors in the long run. Hence, investors who invest in these constituent markets can diversify their portfolio and mitigate risk in the shorter term but not in the long-term. *Fourth*, our findings provide recommendations and implications for regulators and policymakers, as well as cryptocurrency inventors, in developing the foundation for deeper financial integration and supporting a greener business, and eventually society.

The paper is organized into six sections: Sect. 2 describes a concise literature review highlighting the interconnectedness among green bonds, energy markets and cryptocurrencies. Section 3 discusses time-series data and empirical models to be used in the research. Section 4 confers the observed outcomes, Sect. 5 presents the conclusion and Sect. 6 discusses the implications and limitations of study.

## Review of past studies

The asset allocation tactics of investors are linked with the interconnectedness amongst the financial assets, and they frame strategies for diversification (Le et al., 2021). In other words, the different markets worldwide are integrated and have cross-market influences; based on this, investors frame their investment strategies. Research has shown that gold and crude oil have dynamic linkages with other financial assets among varied asset classes. Ciner et al. (2013) concluded that gold could be a secure and the best investment alternative for equities and bonds. The studies also confirm the dynamic relationship with other commodities, such as natural gas and crude oil (Singhal & Ghosh, 2016). Naeem et al., (2021a, 2021b) prove that gold and silver are inextricably linked to the rest of the financial markets. In terms of portfolio diversification in Industry 4.0, fintech and cryptocurrencies and alternative investment, green bonds, are gaining traction among investors. Hence, the literature for such linkages is in three aspects.

First, Fintech companies have arisen as start-ups providing an alternate basis of financial services for lenders, such as crowdfunding, supplier finance, and peer-to-peer lending. These application-based businesses have increased rivalry and competencies and are eventually earning additional profits than conventional financial services (Le et al., 2021). Second, another set of studies focussed on the connectedness of cryptocurrencies with other financial, commodities and energy markets and how this digital platform can be used as a hedging strategy for investment. A recent study by Le et al. (2021) examined the dynamic linkages and frequency domain connectedness between cryptocurrencies and different commodities and energy markets. The study confirms that holding such assets for a longer tenure will lessen the risk. Interestingly, Bitcoin transactions consume high power, which raises environmental and sustainable issues for economies, and therefore policymakers have raised concerns about a resilient economy. Henceforth Naeem and Karim (2021) examined the connectedness between cryptocurrency and the green market and confirmed that clean energy is a valuable hedge for bitcoin. Similarly, Guidici and Polinesi (2021) investigated the effect of price information is transmitted amongst the bitcoin markets and found the strong correlations between different bitcoin prices.

With the mounting gravity of sustainable development goals (SDGs), the policymakers and academicians attempted to explore its nexus in every domain of management and science. It is relatively visible that research on the green bond has gained consideration (Naeem et al., 2021a, 2021b). However, there are two sets of bodies on green finance research. First*,* research on the carbon emission in economy and its various drivers (Ahmed & Jahazeb, 2021; Qin et al., 2021). Further, Flammer (2021) demonstrated that green bonds are efficient at enhancing the sustainable financial performance of organizations. Second*,* Financial analysts and researchers are constantly on the lookout for assets that can serve as a haven and the best diversification strategy during distress. Various authors have studied green bonds as a financial asset as to how they receive or transmit shocks from multiple other markets, their dynamic linkages, and their connectedness with other financial and commodities markets (Le et al., 2021; Naeem et al., 2021a, 2021b; Roboredo, 2018; Roboredo and Ugolin, 2020; Tiwari et al., 2022).

Sustainable energy stocks and the use of green bonds for diversifying the investor’s asset allocation have been emphasized in many studies, however, dynamic linkages of green bonds and renewable energy with cryptocurrencies and their impact on the environment are not robustly studied. In a recent study by Syed et al. (2022) authors examined the asymmetric relationship between economic policy uncertainty, green bonds, and cryptocurrencies, and using NARDL approach authors confirm the existence of an asymmetric association between green bonds, bitcoins, and crypto. Kamal and Hasan (2022) investigated the impact of the popularity of cryptocurrency on clean energy and green assets using quantile-based regression, and quantile connectedness and confirmed the positive effect of cryptocurrency on equity stocks while in the significant relationship with clean energy stocks and green assets. Using a similar methodology of quantile connectedness (Khalfaoui et al., 2022) examined the connectedness between Bitcoins, green markets and economic ambiguity and discovered that global carbon acts as a net receiver of information spillover whereas green assets are the net transmitter of risk. On a similar note, Cai et al. (2021) found the effect of price explosivity on the returns of Bitcoin. Tiwari et al. (2022) investigated the interconnectedness between green bonds and energy markets, confirming that clean energy governs the remaining markets and spreads shocks throughout the system. Additionally, it is found that green bonds and active global wind emerging are the primary receivers of shocks. Polat and Gunay (2021) examined the volatility connectedness between major cryptocurrencies based on market capitalization. The results discovered that connectedness between cryptocurrencies was strong during the crisis. A shred of empirical evidence provided by Braga and Grass (2021a; b) states that policymakers can reduce the risk in green investments by issuing government green bonds compared to private green bonds that exhibit higher volatility in the market. The time-varying spillover between global energy markets was studied using (Baruník & Křehlík, 2018) and the wavelet coherence method by Mensi, Naeem et al. (2021a; b). The authors concluded that spillovers are agile and receptive. Pham (2021) conducted another study on the relationship concerning green bonds and green equity under different market scenarios. Green bonds and green equity have weak connectedness in steady market conditions but strong connectedness during extreme market conditions, according to frequency connectedness and cross-quantilogram methods. The author discovered that the consequences of spillover between green bonds and green equity are transient due to the extent of connectedness fading over moderate and longer timelines.

Similarly, Le et al. (2021) studied the time-varying linkages of green energy with technology markets in industry 4.0, where authors discovered that connectedness between these markets and common is exceptionally high. High risk in the short-run period than in the long-run period indicated the probable losses in the volatile economy. Subsequently, A wide range of studies has been conducted to investigate the relationship betweenclean energy stock with oil prices (Henriques, 2008; Kumar et al., 2012a, b; Ahmad, 2017). These studies confirmed the more considerable impact of technology stocks, in contrast, to clean energy stocks on clean energy markets. Soaring US dollar value enhances the diversification opportunity of green bond investments for market participants in the financial, energy, and commodity markets. Therefore, green and conventional bonds have a stronger association than green bonds, and energy commodities and stock markets have a weak correlation (Reboredo, 2018). Furthermore, various key studies in this area are mentioned in Table 1.

**Table 1:** Key studies on volatility connectedness. *Source*: Author(s) compilation

Source	Objective of Study	Variables	Methods used	Findings
Mzoughi et al. (2022)	Examined the risk transmission between green assets and energy commodities	Green Bonds and energy commodity	FIGARCH Model	Green assets are affected by price spillovers from energy commodities
Ren et al. (2022)	Examined relationship between carbon futures and green bonds	carbon futures and green bond index	Maximum overlap discrete wavelet transforms (MODWT)	Carbon price mostly positively affects green bonds
Attarzadeh and Balcilar (2022)	Connectedenss between among clean energy, Bitcoin, stock market and crude oil	S&P 500, Bitcoin, The Wilder Hill Clean energy index and WTI crude oil prices	TVP-VAR and Diebold and Yilaz (2014)	Connectnedness strengthens during the crisis period
Ren et al. (2022)	Examining the role of clean assets against two distinct cryptocurrencies	Bitcoin, Ethereum, Bitcoin cash, Ethereum classic, Litcoin as dirty cryptocurrencies and Cardano, Ripple, IOTA, Stellar, and Nano	DCC-GARCH Model Diebold Yilmaz (2012)	Dirty cryptocurrencies may find a safe harbour in clean energy
Afzal and Sajeev (2022)	Interconnection between cryptocurrencies and energy market	Bitcoin, Bitcoin Cash, Ethereum, Ripple XRP and Litecoin'sNifty Energy Index, S&P 500 Energy Index, S&P/TSX Canadian Energy Index and Shanghai Stock Exchange Energy Index	Granger Causality and DCC-MGARCH	Overall, there is a modest and poor time-varying link between cryptocurrencies and energy markets
Naeem et al. (2022)	to determine whether bond markets provide hedging for cryptocurrency uncertainty indexes	three bond markets (BBGT, SPGB and SKUK) for three uncertainty indexes of cryptocurrencies (UCRPR, UCRPO and ICEA)	AGDCC-GARCH	Except for SKUK, which is a safe haven investment for cryptocurrency indexes, bond markets are neither hedges nor safe havens
Le et al. (2021)	Connectedness between Fintech, Green bond and cryptocurrency	Fintech, Green bond and cryptocurrency	Diebold Yilmaz (2012) and Baruník and Křehlík (2018)	Traditional assets and modern are good hedgers while fintech stocks are not
Hung (2021)	Investigates the interdependence of green bonds and traditional asset classes	Bitcoin price, S&P 500, Clean Energy Index, and Goldman Sachs Commodity Index (GSCI)	DCC GARCH model	Green bonds and other assets are subject to conditional time-varying reliance, which is low
Huynh et al. (2020)	Examine the connectedness between role of AI in green bond and crytocurrencies	Artifical Intelligence, Green Bond and Bitcoin	Generalized Forecast Error Variance Decomposition	Bitcoin and Gold are important assets for hedging

The literature on investment diversification emphasizing the dynamic linkages of sustainable assets with traditional financial assets, energy, and stock markets reported erratic spillover effects from the conventional bond market to the green bond market (Pham, 2016a, 2016b). Broadstock and Cheng (2019) investigated the dynamic linkages between green and black bonds using dynamic model averaging (DMA). They concluded that these markets are susceptive to financial market motility, economic apprehensions, and investors' spirit towards green bonds. On the other hand, Reboredo et al. (2020) applied structural vector autoregressive (VAR) to the connectedness between varied financial markets and green bonds. The authors concluded a strong connection between currency and fixed income markets and the green bond market, while the relationship is not the same as the stock market. Based on the above extant literature, it can be inferred that green bonds are firmly linked to fluctuations in the traditional bond market. The majority of preceding studies indicate a link between green bonds and financial markets, such as conventional bonds, stock markets and energy markets. However, no research has been conducted to explore the volatility spillover amongst green bonds, energy markets, and cryptocurrency. The current study attempts to add value to the literature by examining volatility spillover in the green bond, energy markets, and cryptocurrency.

The literature on investment diversification by considering the dynamic linkage between green bonds and traditional bonds, share, and energy markets (Pham, 2016a, 2016b) reported changeable spillover effects of the conventional bond market to the sustainable bond market. A piece of thought was revealed by Broadstock and Cheng (2019), who applied dynamic model averaging (DMA) to study the linkage between green and black bonds, and it was discovered that these are delicate to fluctuations in financial market instability, economic policy ambiguity, oil prices, and investors' sentiment towards green bonds (Reboredo et al., 2020). Using the structural vector autoregressive (VAR) model to figure out the connectedness between financial markets such as sustainable bond markets confirms the strong connection between the financial market and green bond market. At the same time, the relationship is not the same with the stock market. Hence, with the above string of literature, it can be easily understood that green bonds are firmly linked to fluctuations in the traditional bond market. Nevertheless, they are poorly associated with risky energy commodities and stock markets. The above research findings do not present substantial evidence of volatility amongst the green bond, energy market, and cryptocurrency.

The majority of preceding studies indicated robust evidence of the interconnectedness of green bonds with financial markets, such as traditional bonds, stock markets and energy markets. One set of literature examines the interconnectedness between the green bond and other commodities, while another strand of literature covers the association of green bond with renewable energy stock. However, there is a dearth of studies exploring the volatility spillover amongst the green bonds, energy markets, and technology markets such as cryptocurrency. The research literature on cryptocurrencies is still in its early stages, but rapidly expanding (Giudici & Polinesi, 2021). Hence, this study attempts to bridge the gap by examining volatility spill over from green bond to energy and crypto market.

## Data and econometrics

### Data

**Fig. 1:**
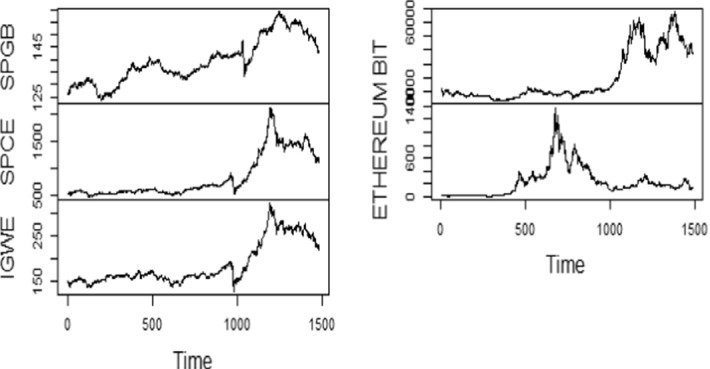
Time series plot-raw series. *Source*: Author(s) calculations

**Fig. 2:**
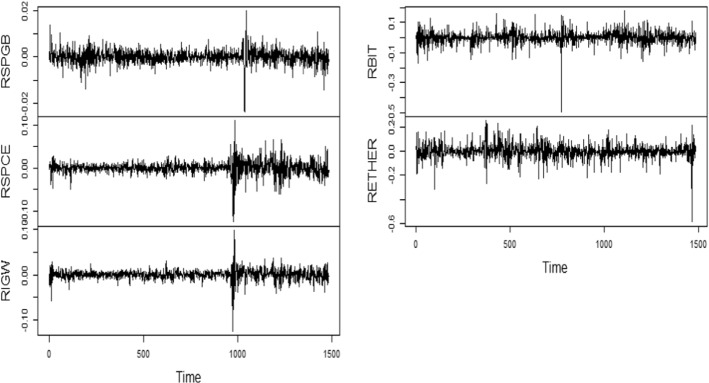
Time series plot-return series. *Source*: Author(s) calculations

This paper attempts to unravel the dynamic linkages of green bond with energy and crypto market. To measure the green bond, S&P green bond index (RSPGB) is considered, while S&P global clean energy index (RSPCE) and ISE global wind energy (RIGW) signifythe renewable energy market. Further, Bitcoin (RBIT) and Ethereum (RETHER) are taken as proxies of crypto market. These proxies are used in Tiwari et al. (2022), Naeem et al. (2021a; b), Abakah et al. (2021). We collect the daily prices of these constituent series from Bloomberg, extending from October 3, 2016 to February 23, 2021. Each series is converted into a log return differencing the log of the concerned series. Table 2 encapsulates the data description of the series considered under examination. To check the pattern, we report the graphical depiction of raw and log return series in Figs. 1 and 2.

**Table 2:** Data description of the constituent markets. *Source*: Author’s own presentation

Asset/index	Proxy	Abbreviation	Description
Green bond	S&P Green bond index	RSPGB	Tracks and measures the effectiveness of labeled green bonds
Energy market	S&P global clean energy index	RSPCE	Evaluates the efficiency of businesses in international clean energy in emerging and established economies
	ISE global wind energy index	RIGW	Tracks performance of companies that are involved in the wind industry
Cryptocurrency	Bitcoin	RBIT	It decentralized digital currency without the need for any intermediaries
	Ethereum	RETHER	Decentralized open-source digital currency, second after Bitcoin in terms of market capitalization

Figure 1 exhibits the intense fluctuations in raw series, especially under green bonds throughout the period. Fascinatingly, there has been tremendous momentum seen in all variables, exclusively in Bitcoin and renewable energy, during the recent years, which is the period of the COVID-19 outbreak. Further, Fig. 2 exhibits the multiple volatility clusters for all series during the study period.

### Econometric models

The dynamic conditional correlation (DCC), Diebold and Yilmaz (2012), and Baruník and Krehlik (2018) models are employed to investigate the dynamic linkages of green bonds with the energy and crypto markets. The details of these models are discussed below:

#### Dynamic conditional correlation (DCC)

We employ the DCC-GARCH model pioneered by Engle (2002) to test the time-varying correlations/dynamic linkages of green bond with renewable energy and the crypto market. This model is highly preferred for several reasons; first, it identifies dynamic investor behavior against current news and events by detecting possible changes in conditional correlations over time (Celik, 2012). Second, Due to increased dimensionality in the assets and increased volatility, constant correlations arise, which is the basic assumption of the basic GARCH model; therefore, this DCC-GARCH model relaxes this assumption and provides for time-varying correlations (Singhal & Ghosh, 2016). Third*,* The DCC-GARCH model calculates correlation coefficients from standardised residuals and accounts for heteroskedasticity (Chiang et al., 2007). This model corrects the correlations for time-varying volatility, making it the only correlation measure (Cho & Parhizgari, 2008). The DCC-GARCH model is computed in two stages: (1) estimation of the univariate GARCH modeland (2) the conditional correlations.

The multivariate DCC-GARCH model is expressed mathematically:

1 $$ X_{t} = u_{t} + H_{t}^{1/2} \varepsilon_{t} $$

2 $$ \left\{ \begin{gathered} H_{t} = D_{t} R_{t} D_{t} \hfill \\ R_{t} = \left( {diag(Q_{t} )} \right)^{1/2} Q_{t} \left( {Diag(Q_{t} )} \right)^{ - 1/2} \hfill \\ D_{t} = diag\left( {\sqrt {h_{11,t} } ,\sqrt {h_{22,t} } , \ldots ,\sqrt {h_{NN,t} } } \right) \hfill \\ \end{gathered} \right\} $$

where $X_{t} = (X_{1t} ,X_{2t} ,X_{3t} , \ldots X_{Nt} )$ is the vector of previous observations $H_{t}$ is the multivariate conditional variance. The vector of conditional returns is $u_{t} = (u_{1t} ,u_{2t} ,u_{3t} , \ldots u_{N,t} )$ and the vector of standardized results is $\varepsilon_{t} = (\varepsilon_{1t} ,\varepsilon_{2t} , \ldots \varepsilon_{Nt} )$ and D_t_ is a diagonal matrix of conditional standard deviation for *R*_*t is*_ an return series NXN symmetric dynamic correlations matrix, derived from univariate GARCH model with $\sqrt {h_{ii,t} }$, i = 1, 2,…N diagonals.

The DCC specification is as follows:

3 $$ \begin{aligned} & Q_{t} = \left( {1 - \psi - \zeta } \right)\overline{Q} + \zeta Q_{t - 1} + \psi \delta_{i,t - 1} \delta_{j,t - 1} \\ & R_{t} = Q_{t}^{* - 1} Q_{t} Q_{t}^{* - 1} \\ \end{aligned} $$

where $(Q_{t} ) = \left| {q_{ij,t} } \right|$ is (NXN) time varying covariance normalised residual matrix of $\left( {\delta_{it} = \frac{{\varepsilon_{it} }}{{\sqrt {h_{it} } }}} \right)$, $\overline{Q}$ is the unconditional correlations of $\delta_{i,t} ,\delta_{j,t} \;and\;\psi ,\varsigma$ are non-negative scalar parameters that satisfies $\psi + \varsigma (1.Q_{t}^{*} = \left[ {q_{ii,t}^{*} } \right] = \sqrt {q_{ii,t} }$ is a diagonal matrix containing square root of the ith diagonal element of *Q*_*t*_ on its ith diagonal position.

As a result, the conditional correlation at time t for a set of markets *i* and *j*is defined below:

4 $$ \rho_{ij,t} = \frac{{\left( {1 - \psi - \varsigma } \right)\overline{q}_{ij} + \psi \delta_{i,t - 1} \delta_{j,t - 1} + \varsigma q_{ij,t - 1} }}{{\left[ {(1 - \psi - \varsigma )\overline{q}_{{_{ii} }} + \psi \delta_{i,t - 1}^{2} + \varsigma q_{ii,t - 1} } \right]^{1/2} \left[ {(1 - \psi - \varsigma )\overline{q}_{jj} + \psi \delta_{j,t - 1}^{2} + \varsigma q_{jj,t - 1} } \right]^{1/2} }} $$

where q_*ij*_ is the element on the *i*th line and *j*th column of the matrix Q_t_, Bollerslev et al. (1992) devised the quasi-maximum likelihood technique (QMLE) for estimating the parameters.

The estimator log-likelihood under the Gaussian assumption is:

5 $$ L(\vartheta ) = - \frac{1}{2}\sum\limits_{t = 1}^{T} {\left[ {\left( {\left( {n\log 2\pi } \right) + \log \left| {d_{t} } \right|^{2} + \varepsilon_{t}^{^{\prime}} D_{t}^{ - 1} D_{t}^{ - 1} \varepsilon_{t} } \right) + \left( {\log \left| {R_{t} } \right| + \delta_{t}^{^{\prime}} R_{t}^{ - 1} \delta_{t} \delta_{t}^{^{\prime}} \delta_{t} } \right)} \right]} $$

where n = the no. of equations, T = no. of observations, and $\vartheta$ = vector of parameters to be estimated.

#### Diebold and Yilmaz (2012) model

To refine the dynamic linkages, Diebold and Yilmaz (2012) Model is employed, which is based on the dynamic forecast error variance decomposition within the vector autoregressive (VAR) framework (Koop et al., 1996). This technique is highly preferred over the previous spillover techniques because, first*,* because this technique ignoresthe VAR’SCholesky factor identification, the findings are unaffected byorder of the variables (Naeem et al., 2021a; b). Second, it consents to trace the connectedness at different levels (Le et al., 2021). As a result, it overcomes the problem of time variation by becoming a dynamic spillover model. The goal of this model is to examine the spillover contributions of “TO” and “FROM” other variables in the model using the VAR model’s comprehensive and intuitive forecast error variance decomposition (Abbas et al., 2019). Moreover, this model also captures the net transmission or receiver at a specific stretch of time. The specification of model consider period, *t* = 1,… *T*, structured VAR(*p*) with n-variate method *X*_*t*,1_,…* X*_*t,n*_ presented below:

6 $$ \Upsilon \left( L \right)x_{t} = \varepsilon_{t} $$

where $\Upsilon \left( L \right) = \mathop \sum \limits_{h} \Upsilon_{h } L^{h}$ denotes a n × m coefficient matrix *t* by means of an infinity-length lag polynomial. Consequently, the FEVD, is consistent alongside Diebold and Yilmaz (2012), and is shown below:

7 $$ \left( {\theta_{H} } \right)_{j,k} = \frac{{\sigma_{{kk\mathop \sum \nolimits_{h = 0}^{H} \left( {{\Psi }_{h } \sum } \right)\left( {\emptyset_{h} \sum } \right)\left( {{\Psi }_{h } \sum } \right)j,k)2}}^{ - 1} }}{{\mathop \sum \nolimits_{h}^{H} \left( {{\Psi }_{h } \sum {\Psi }_{h}^{^{\prime}} } \right)_{jj} }} $$

where $\sigma_{kk } = \left( \in \right)_{k,k. }$ and $\emptyset_{h }$ signifies a n × m matrix having $lag h.\left( {\theta_{H} } \right)_{j,k}$ elucidates that *k*th variable is responsible for tremors to the variation of some other parameter forecast error, *j*. On computing, the sum of each row of (θ_H) (*j, k*) does not approximate unity. As a result, the row's sum is utilised to segregate the specific constituent of the decomposition matrix for normalisation. Mathematically, it can be stated as:

8 $$ \left( {\overline{{\theta_{H} }} } \right)_{j,k } = \frac{{\left[ {\theta_{H} } \right]_{j,k} }}{{\mathop \sum \nolimits_{k = 1}^{H} \left( {\theta_{H} } \right)_{j,k} }} $$

with $\mathop \sum \nolimits_{k = 1}^{H} \left( {\theta_{H} } \right)_{j,k} = 1 \;and\; \mathop \sum \nolimits_{j,k = 1}^{H} \left( {\theta_{H} } \right)_{j,k} = {\text{N}}$.As a result, according to DY (2012), the degree of spillover is a part of the combined elements in the off-diagonal total matrix, as under:

9 $$ C_{H} = \frac{{\mathop \sum \nolimits_{j \ne k} \left( {\overline{\theta }_{H} } \right)_{j,k} }}{{\sum \left( {\overline{\theta }_{H} } \right)_{j,k} }} \times \, 100 \, = \left( {1 - \frac{{Tr \left\{ {\overline{\theta }_{H} } \right\}}}{{\sum \left( {\overline{\theta }_{H} } \right)_{j,k} }}} \right)100 $$

where C_H_ represents the network's overall spillover, restricted as the proportionate contributor to predicting variances from the residual series in the network, the trace operator stands as Tr{.} The directional spillover acknowledged by a specific parameter, *j*, to other variables *k* in the network can be projected for a more definitive spillover parameter:

10 $$ \left( {C_{H} } \right)_{j \to } = 100 \times \frac{1}{n}\mathop \sum \limits_{j \ne k,k} \left( {\overline{\theta }_{H} } \right)_{j,k} $$

11 $$ \left( {C_{H} } \right)_{j \leftarrow } = 100 \times \frac{1}{n}\mathop \sum \limits_{j \ne k,k} \left( {\overline{\theta }_{H} } \right)_{j,k} $$

where $\left( {C_{H} } \right)_{j \to }$ and $\left( {C_{H} } \right)_{j \leftarrow }$ stands for “TO spillover” and “FROM spillover” correspondingly.

Further, net spillover in case of variable *j* is calculated here as "FROM spillover” deducted from "To spillover" which is mathematically presented as below:

12 $$ \left( {C_{H} } \right)_{j, net } = \left( {C_{H} } \right)_{j \to } - \left( {C_{H} } \right)_{j \leftarrow } $$

Through a positive net spillover value,$\left( {C_{H} } \right)_{j, net } ,$ infers that the variable *j *depicts the net shock transmitter. Variable *j* is a shock recipient if the coefficient is lower (negative).

#### Baruník and Křehlík (2018) model

In their mathematical framework for spillover, Baruník and Křehlík (2018) demonstrates the interconnectedness of variables that fluctuate at various wavelengths by modifying the DY spillover framework (2012). Based upon that affirmation in Eq. (4), which implies that the impulse function h is time-varying, another approach assumes the impulse function term reflecting the bandwidth. In crux, the frequency response function converts to ${\Psi }\left( {e^{ - iw} } \right) = \mathop \sum \limits_{h} e^{{ - iwh\emptyset_{h} }}$, obtained from ${\Psi }$ capturing the coefficients of the fourier transform, with $i = \sqrt { - 1}$. The generalised causation spectrum across the identified frequencies ω =  ∈ (− π, π) is expressed as follows:

13 $$ \left( {f(\omega )} \right)_{j,k} \equiv \frac{{\sigma_{kk}^{ - 1} \left| {\left( {{\Psi }(e^{ - iw} } \right)\sum_{j,k} } \right|2}}{{\left( {{\Psi }\left( {e^{ - iw} } \right)} \right)\sum {\Psi }^{{\prime }} \left( {e^{ + iw} } \right)_{jj} }} $$

where $\emptyset \left( {e^{ - iw} } \right)$ represents the impulse response’s frontier transform $\emptyset . \left( {f\left( \omega \right)} \right)_{j,k}$ representsthe proportion of the jth variable’s spectrum at frequency $\omega$, induced by shocks in the kth variable. $\left( {f\left( \omega \right)} \right)_{j,k}$ is understood as a measure of within-frequency causation. As a result, of Baruník and Křehlík (2018), the generalized FEVD (GFEVD) on a particular frequency bandwidth d is here figured as under:

14 $$ \left( {\theta_{d} } \right)_{j,k } = \frac{1}{2\pi }\mathop \smallint \limits_{{}}^{d} {\Upsilon }_{j} \left( \omega \right)\left( {f\left( \omega \right)} \right)_{j,k} d\omega $$

The weighing function is denoted by ${\Upsilon }_{j} \left( \omega \right)$. Considering the spatial depiction of the GFEVD, the frequency-based interconnectedness on the frequency wavelength is described as:

15 $$ C_{d}^{F } = 100 \left( {\frac{{\sum_{j \ne k } \left( {\overline{\theta }_{d} } \right)j,k}}{{\sum \left( {\overline{\theta }_{\infty } } \right)_{j,k} }} - \frac{{Tr\left\{ {\overline{\theta }_{d} } \right\}}}{{\sum \left( {\overline{\theta }_{\infty } } \right)_{j.k} }}} \right) $$

As a result, the total spillover is calculated as follows:

16 $$ C_{d}^{w} = 100\left( {1 - \frac{{Tr\left\{ {\overline{\theta }_{d} } \right\}}}{{\sum \left( {\overline{\theta }_{d} } \right)_{j,k} }}} \right) $$

Directional spillovers, like time-domain spillovers, indeed be estimated at diverse wavelengths. "TO," "FROM," and "net" spillover now be computed in Eqs. (12)–(14), which are corresponding to Eqs. (5) and (7).

17 $$ (C_{d}^{F} )_{j \to } = 100 \times \left( {\mathop \sum \limits_{j \ne k,k} \left( {\overline{\theta }_{d} } \right)_{j,k} } \right)\frac{{\sum \left( {\overline{\theta }_{d} } \right)_{j,k} }}{{\sum \left( {\overline{\theta }_{\infty } } \right)_{j,k} }} $$

18 $$ (C_{d}^{F} )_{j \leftarrow } = 100 \times \left( {\mathop \sum \limits_{j \ne k,k} \left( {\overline{\theta }_{d} } \right)_{j,k} } \right)\frac{{\sum \left( {\overline{\theta }_{d} } \right)_{j,k} }}{{\sum \left( {\overline{\theta }_{\infty } } \right)_{j,k} }} $$

19 $$ (C_{d}^{F} )_{j, net } = (C_{d}^{F} )_{j \to } - (C_{d}^{F} )_{j \leftarrow } $$

A positive value of $(C_{d}^{F} )_{j, net}$ means that variable under question isa net transceiver of disruptions to other parameterswhile a negative value signifies a net recipient.

## Empirical results and discussion

In this section, we discuss the results obtained from summary statistics, unit root, dynamic conditional correlation (DCC-GARCH), Diebold and Yilmaz (2012) and Baruník and Křehlík (2018), which examines the return transmission mechanism of green bond with energy and crypto-market.

### Summary statistics and unit root testing

To investigate the spillover effect between the green bond, clean energy and cryptocurrency market, we applied the dynamic conditional correlation model. Moreover, the interconnectedness between the variables in different time horizons have been studied by applying the Diebold and Yilmaz (2012) and Baruník and Křehlík (2018) model. We begin an analysis presenting the outcome of descriptive statistics, which is shown in Table 3. The mean returns for all variables are positive; the highest mean can be seen for cryptocurrencies, RETHER (0.0017) and RBIT (0.0008), followed by clean energy markets RPSCE (0.0005), Wind energy market RIGW (0.0003), and green bond RSPGB (0.0001). Thus, the green bond has the lowest mean return, but it also comparatively has the lowest standard deviation. In skewness estimates, the series are moderately left-skewed with a leptokurtic distribution, indicating that the series are statistically not normally distributed; the Jarque–Bera test confirms the same. On a final note, Augmented Dickey Fuller (ADF) test and Phillips and Perron (PP) test signify that all series are stationary and exhibit ARCH effect at 0.01 and 0.05 level of significance. ADF is the most widely used method for determining time-series data stationarity (Yadav et al., 2021). Hence, dynamic conditional correlation (DCC) is applied in the next step to examine the dynamic linkages.

**Table 3:** Summary statistics of constituent series

	RSPGB	RSPCE	RIGW	RBIT	RETHER
Minimum	− 0.0241	− 0.1250	− 0.1259	− 0.4973	− 0.5896
Maximum	0.0201	0.1103	0.0989	0.1774	0.2586
Mean	0.0001	0.0005	0.0003	0.0008	0.0017
SD	0.0030	0.0152	0.0113	0.0402	0.0598
Skewness	− 0.6852	− 0.7934	− 1.0761	− 1.3318	− 0.6518
Kurtosis	8.4374	11.5006	17.9314	17.4085	8.9736
Jarque–Bera test	0.0000***	0.0050***	0.0001***	0.0000***	0.0030***
ADF-test	0.0000***	0.0100**	0.0001***	0.0000***	0.0000***
PP Test	0.0020**	0.0000***	0.0000***	0.0000***	0.0000***
ARCH Test	0.0000***	0.0000***	0.0100**	0.0000***	0.0000***

** and *** Indicates the significance level at 1% and 0.01% respectively. Jarque and Bera (1980) is conducted for checking normality of the data. Augmented Dickey et al. (1984) and Phillips–Perron test are used to check the stationarity of the respective variables

Furthermore, we can better comprehend how the series changed over as represented in Figs. 1 and 2. Volatility clustering can be seen in every series, where high changes are followed by high changes and low changes are followed by low changes.

### An insight of dynamic conditional correlation

Further, spillover is then examined using dynamic conditional correlation generalized Autoregressive conditional heteroskedasticity (DCC-GARCH) model. Bivariate dynamic conditional correlation (DCC) GARCH, as shown in Table 4, was used for the statistical analysis. The table consist the spillover results from green bond market (RGPGB) to global clean energy market (RSPCE), global wind energy market (RIGW), Bitcoin (RBIT) and Ethereum (RETHER). We report the results obtained from DCC to represent the dynamic linkages of green bond (RSPGB) with energy market (RSPCE, RIGW) and crypto-market (RBIT, RETHER) in Table 4(A–D), respectively. In these tables, ‘mu’ represents the overall mean and ‘const’ represents the intercept term. Furthermore, α (ARCH) represents the effect of previous disturbances or the error term obtained through the mean equation, whereas β (GARCH) represents the effect of the last variance. DCC generates the results in the form of univariate and bivariate GARCH: first, it means ARCH and GARCH terms; second, it provides DCCa1 and DCCb1 (Sharma et al., 2021). Considering the univariate GARCH, it is noticed that α 1 and β 1 of green bond (RSPGB) and Cryptocurrency (RBIT) remains insignificant statistically while rest are significant. It signifies that new information is not captured in green bond and bitcoin. Further, each series has beta 1, displaying volatility persistence. The summation of alpha 1 and beta 1 is less than 1 required in univariate GARCH. The coefficient is less than 1 refers to non-stationarity in decaying the volatility.

**Table 4:** DCC of green bond with renewable energy and cryptocurrency

Series	Coefficients	Standard error	T-value	*P* value
*Panel A: DCC from RSPGB to RSPCE*				
[RSPGB] mu	0.0001	0.0001	1.7838	0.0745
[RSPGB] const	0.0000	0.0000	0.2652	0.7908
[RSPGB] α	0.0633	0.0345	1.8341	0.0666
[RSPGB] β	0.9123	0.0331	27.5911	0.0000***
[RSPCE] mu	0.0006	0.0003	2.2249	0.0261*
[RSPCE]const	0.0000	0.0000	0.8023	0.4224
[RSPCE] α	0.1265	0.0495	2.5570	0.0106*
[RSPCE] β	0.8681	0.0467	18.6078	0.0000***
[Joint]dcca1	0.0018	0.0068	0.2571	0.7971
[Joint]dccb1	0.9503	0.0343	27.6723	0.0000***
*Panel B: DCC from RSPGB to RIGW*				
[RSPGB]mu	0.0001	0.0001	1.9270	0.0540
[RSPGB]const	0.0000	0.0000	0.3213	0.7480
[RSPGB] α	0.0684	0.0345	1.9853	0.0471*
[RSPGB] β	0.9040	0.0333	27.1678	0.0000***
[RIGW]mu	0.0005	0.0002	2.3148	0.0206*
[RIGW]const	0.0000	0.0000	0.2287	0.8191
[RIGW] α	0.1493	0.0270	5.5376	0.0000***
[RIGW] β	0.8188	0.1355	6.0417	0.0000***
[Joint]dcca1	0.0000	0.0000	0.0000	1.0000
[Joint]dccb1	0.9149	0.1227	7.4561	0.0000***
*Panel C: DCC from RSPGB to RBIT*				
[RSPGB]mu	0.0001	0.0001	2.0547	0.0399*
[RSPGB]const	0.0000	0.0000	0.2113	0.8327
[RSPGB] α	0.0647	0.0411	1.5756	0.1151
[RSPGB] β	0.9125	0.0388	23.5032	0.0000***
[RBIT]mu	0.0014	0.0010	1.4022	0.1609
[RBIT]const	0.0001	0.0001	2.1481	0.0317*
[RBIT]α	0.0771	0.0413	1.8674	0.0618
[RBIT] β	0.8549	0.0472	18.0940	0.0000***
[Joint]dcca1	0.0022	0.0097	0.2228	0.8237
[Joint]dccb1	0.9200	0.1143	8.0499	0.0000***
*Panel D: DCC from RSPGB to RETHER*				
[RSPGB]mu	0.0001	0.0001	2.1029	0.0355
[RSPGB]const	0.0000	0.0000	0.2776	0.7813
[RSPGB] α	0.0671	0.0371	1.8073	0.0707
[RSPGB] β	0.9077	0.0350	25.9363	0.0000***
[RETHER]mu	0.0010	0.0013	0.7327	0.4637
[RETHER]const	0.0003	0.0001	3.0798	0.0021**
[RETHER] α	0.1663	0.0395	4.2072	0.0000
[RETHER] β	0.7568	0.0437	17.3268	0.0000***
[Joint]dcca1	0.0054	0.0113	0.4794	0.6316
[Joint]dccb1	0.9040	0.0509	17.7574	0.0000***

RSPGB signifies the S&P Green bond index, RSPCE signifies S&P global clean energy market, RIGW signifies global wind energy, RBIT signifies Bitcoin and RETHER signifies Ethereum

dcca1 signifies the spillover in the short-run, dccb1 signifies spillover in long-run

*, ** and *** Indicate the significance level at 5%, 1% and 0.01% respectively

**Table 5:** Connectedness using Diebold Yilmaz (2012)

Series	RSPGB	RSPCE	RIGW	RBIT	RETHER	FROM
RSPGB	99.22	0.23	0.09	0.23	0.24	0.16
RSPCE	0.38	88.61	10.67	0.30	0.04	2.28
RIGW	0.35	8.30	91.15	0.10	0.10	1.77
RBIT	0.20	0.19	0.14	99.26	0.22	0.15
RETHER	0.08	0.03	0.05	0.16	99.69	0.06
TO	0.20	1.75	2.19	0.16	0.12	4.42
Net	− 0.04	0.53	− 0.42	− 0.01	− 0.06	

This table displays the result obtained from DY approach, considering volatility spillover of various markets considered under examination. The values in the *i*th row of the *j*th column indicate the strength of the spillover effect from the *i*th market to the *j*th market

Regarding bivariate dynamic conditional correlation (bivariate GARCH), dcca1 and dccb1 depict the spillover or dynamic linkages of one market with another in the short and long run respectively (Yadav et al., 2020). As per panel 4(A), dcca1 is positive and insignificant, which implies no integration or spillover of green bond with energy market (RSPCE) in the short-run. On the contrary, dccb1 is positive and significant, indicating spillover in the long run. Further, in panel 4(B), dcca1 is positive and insignificant, suggesting that there is no spillover between green bond and wind energy in the short-run. In contrast, there is spillover from green bond to wind energy in the long-run as dccb1 is positive and insignificant. The dcca1 in panel 4(C) is positive and insignificant, signifying no short-run spillover from green bond to Bitcoin, whereas positive coefficient and significant p-value of dccb1 indicate spillover from green bond to Bitcoin in long-run. Similarly, in panel 4(D) there is no short-run spillover from green bond to Ethereum. In contrast, the positive coefficient and significant p-value of dccb1 indicate spillover from green bond to Ethereum in the long-run.

**Fig. 3:**
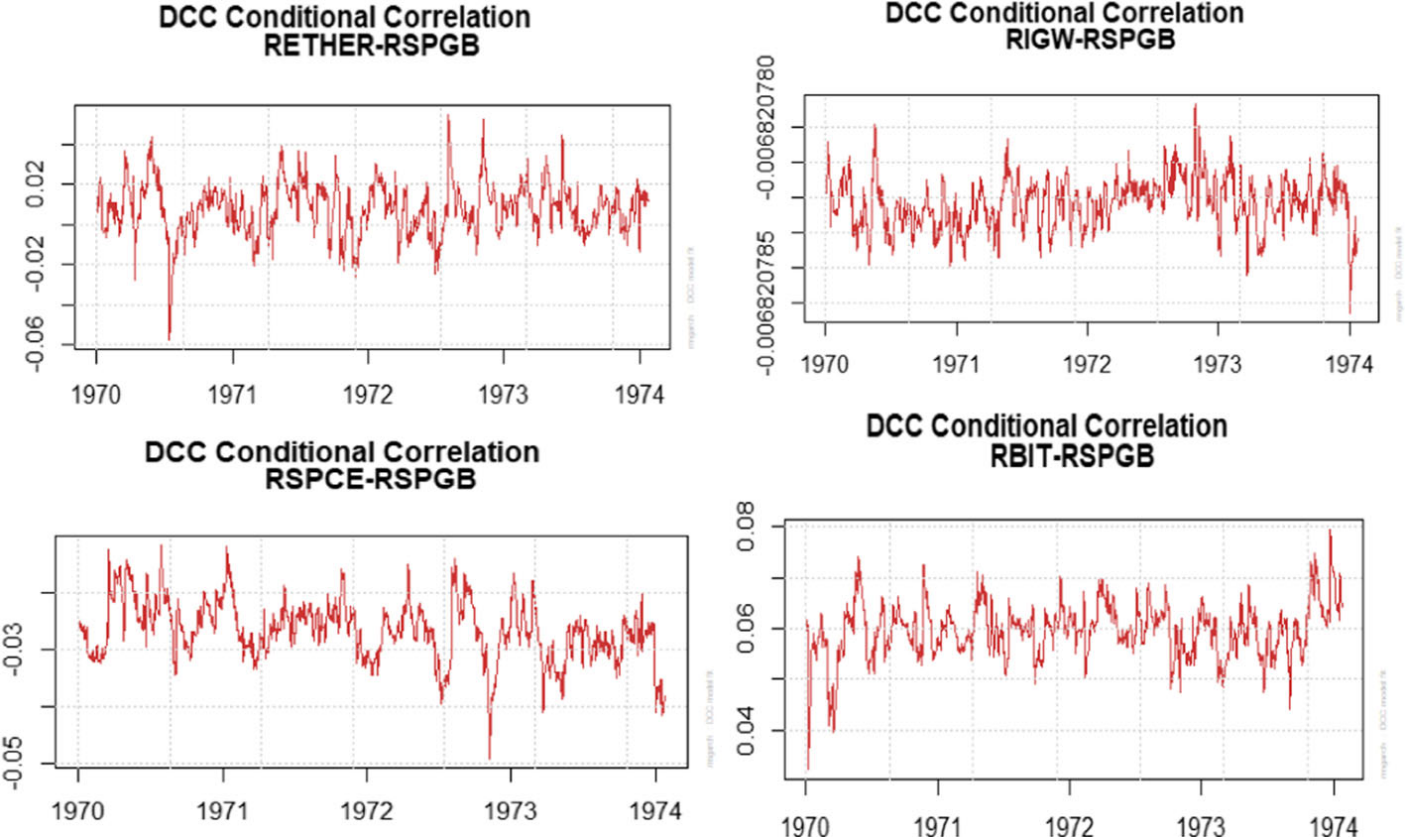
Time varying correlation among constituent series

In summary, our findings demonstrate that there are no dynamic linkages of volatility spillover from green bond to energy and crypto market in the short run. According to the DCC findings, investors who invest in these constituent markets can diversify their portfolio and mitigate risk in the shorter term but not in the long-term. To be precise, the shocks experienced by the green bond market does not affect energy and crypto-market in the short run while it may affect in the long run. Our findings are similar to Roeredo and Ugolini (2020), Pham (2016a, 2016b). Further, Fig. 3 displays the graphical representation of the time-varying correlation of green bond market with the constituent market. We observe that correlation is dynamic and not constant as they vary according to time.

### Evidence of dynamic linkages using Diebold and Yilmaz (2012)

We document the result of Diebold and Yilmaz (2012) for dynamic linkage of green bond with energy and crypto-market in Table 5. It is noticed that DCC shows the spillover in the short and long run, but it does not provide its magnitude. To overcome this, Diebold and Yilmaz (2012) model is employed amongst these markets. In this table, within and cross-market dynamic linkages are presented by the diagonal and off-diagonal elements of the matrix. Similarly, ‘From’ is the average value of connections or connectedness derived from constituent markets while “To” shows the average value of connectedness contributed to other markets.

Regarding volatility transmission received from other markets, we observe that one of the energy market indexes (RSPCE) has the highest linkages derived fromgreen bond and cryptocurrency followed by other index of the energy market (RIGW) that is 2.28% and 1.77% respectively. Similarly, cryptocurrency (RETHER) derives the least volatility spillover (0.06) from other constituent markets. Further, the energy market itself (RIGW) and cryptocurrency (RETHER) are largest and least contributors to the transmission of the volatility. On this note, it can be observed that the energy market is a quick responder of the shocks in terms of both recipient and transmission. Next, we compute the net directional dynamic linkage differentiating between “From” and “To” spillover. It helps to detect which market receives more spillover than it transmits and vice versa (Tiwari et al., 2022). Referring to the net directional dynamic linkage, it is found that green bond, one index of energy market (RIGW) and cryptocurrency (both Bitcoin and Etherum) are net receivers of the shocks with − 0.4, − 0.42, − 0.01 and − 0.06 respectively. It is S&P global clean energy index (RSPCE) which is only transmitter of shocks, hence, it dominates the rest of the constituent assets class.

Additionally, we document the own variable shocks diagonally in the same table and observe that 99.22% of green bond, 88.61% of energy market (RSPCE), 91.5% of energy market (RIGW), 99.26% of cryptocurrency (RBIT), 99.69% of cryptocurrency (RETHER) are propelled by its shocks. On this note, it is witnessed that 11.39% movement in the energy market (RSPCE) is affected by its network connection which is high comparatively. To sum up, we find that green bond (RSPGB) is a net receiver, though the contribution from other markets is less, hence, it can be said that it is marginally connected with energy and crypto market. This weak dynamic linkage appears because of lower degree of competition (Naeem et al., 2021a, 2021b). This evidence is in the similar line with the findings of Roeredo and Ugolini (2020), Pham (2016a, 2016b) and different from Tiwari et al. (2022).

**Fig. 4:**
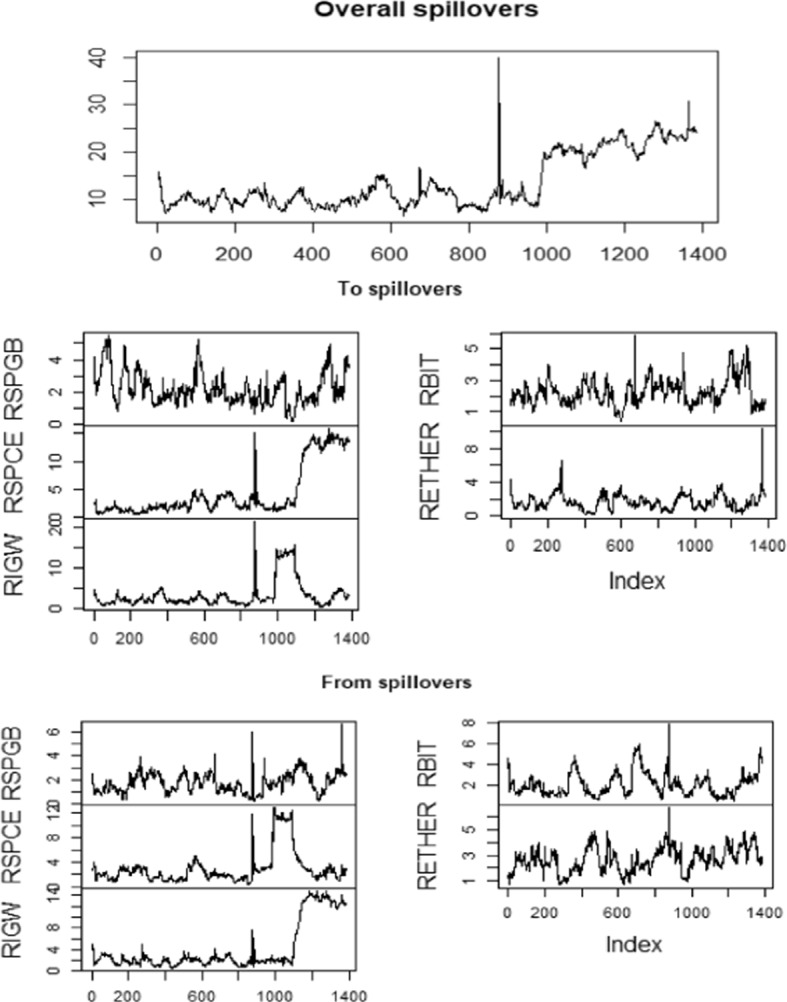
Overall, to and from spillover amongst various markets

Figure 4 displays the graphical representation of overall spillover, from spillover to spillover employing Diebold and Yilmaz (2012). Considering the time aspect, the observation number 0, 200, 400, 600, 800, 1000, 1200 and 1400 denote March 10, 2016, December 21, 2016, September 27, 2017, July 6, 2018, April 16, 2019, January 21, 2020, October 27, 2020 and October 15, 2021 respectively. From this figure, we notice that connectedness among the various markets is varying; however, during the end of 2019, high connectedness is seen in each market except cryptocurrency (RETHER).

### Dynamic linkages using Baruník and Křehlík (2018) model

The dynamic connectedness amongst the market varies over the period because of their pattern and stochastic nature. Hence, we employ the Baruník and Křehlík (2018) model to refine the connectedness in various time horizons. The result is obtained considering 1 day to 4 days (short term frequency connectedness), 4 days to 10 days (medium-term frequency connectedness) and 10 days to infinite days (long term frequency connectedness); the same is presented in Table 6(A–C) respectively. In these tables, the terminologies like “WTH” signifies within, “ABS '' signifies absolute, “FROM” refers to spillover received from other asset classes and “TO” illustrates spillover contributions to markets. Referring to the Table 6(A), we conjecture that the energy market (ISE global wind energy index-RIGW) derives highest spillover from other series (1.50%) followed by another index of energy market (S&P global clean energy index-RSPCE) with 1.41% in short-run. Furthermore, in the medium run, clean energy market is the highest receiver of spillover from other series (3.86%) followed by wind energy (2.24%). Interestingly, wind energy is the highest contributor (3.82%) followed by clean energy (2.14) in the medium-term horizon. Moreover, in the long-run clean energy obtains highest spillover (5.09%) followed by wind energy (2.69%), and in divergent case wind energy is the highest receiver of the spillover from other series. From Baruník and Křehlík (2018) frequency-domain result, total connectedness of the five series is observed to be higher in the long-run than in the short run, thus suggesting reduced diversification opportunities in the long run.

**Table 6:** Application of the Baruník and Krehlík (2018)

	RSPGB	RSPCE	RIGW	RBIT	RETHER	FROM_ABS	FROM_WITH
*(A)*							
RSPGB	65.99	0.21	0.04	0.22	0.09	0.11	0.16
RSPCE	0.28	60.92	4.41	0.26	0.01	0.99	1.41
RIGW	0.13	4.94	61.29	0.10	0.10	1.05	1.50
RBIT	0.12	0.16	0.07	76.34	0.22	0.11	0.16
RETHER	0.07	0.01	0.03	0.05	75.42	0.03	0.05
TO_ABS	0.12	1.07	0.91	0.13	0.08	2.30	
TO_WTH	0.17	1.52	1.29	0.18	0.12		3.28
*(B)*							
RSPGB	20.53	0.01	0.03	0.01	0.09	0.03	0.15
RSPCE	0.08	16.51	3.38	0.02	0.02	0.70	3.86
RIGW	0.12	1.90	17.79	0.00	0.00	0.41	2.24
RBIT	0.05	0.01	0.04	14.52	0.00	0.02	0.11
RETHER	0.01	0.01	0.01	0.06	15.35	0.02	0.09
TO_ABS	0.05	0.39	0.69	0.02	0.02	1.17	
TO_WTH	0.28	2.14	3.82	0.11	0.11		6.46
*(C)*							
RSPGB	12.69	0.00	0.02	0.00	0.06	0.02	0.15
RSPCE	0.02	11.18	2.89	0.01	0.02	0.59	5.06
RIGW	0.10	1.45	12.06	0.00	0.00	0.31	2.69
RBIT	0.04	0.01	0.03	8.40	0.00	0.02	0.14
RETHER	0.00	0.01	0.01	0.04	8.92	0.01	0.10
TO_ABS	0.03	0.29	0.59	0.01	0.02	0.94	
TO_WTH	0.27	2.54	5.08	0.10	0.14		8.13

(**A**) 1–4 days (band 3.14–0.79), (**B**) 4–10 days (band 0.79–0.31), (**C**) 10 days to inf days (band 0.31–0). Source: Author(s) calculations

The primary reason for the high spillover in the long run, is that it takes time for any event to reflect its impact. Furthermore, long-term investors are concerned about the energy-efficient green bond markets to provide investment alternatives for investors. Due to this,a demand and supply mismatch problem occurs, and its impact can be seen in the long run (Sharma et al., 2021; Naeem et al., 2021a, 2021b).

## Conclusion

Past studies confirmed that using cryptocurrency in a portfolio together with traditional assets has the advantage of increasing diversification (Moreno et al., 2022). Sustainable economic development and climate change have caught the attention of global leaders for fighting overall environmental degradation and the adoption of green bonds. Similarly, renewable energy, which includes water, biomass, wind, solar, and wave energy, provides an alternative to fossil fuels to reduce carbon emissions for a healthy economy. Regardless of other environmental penalties, Bitcoin and other cryptocurrencies could drive global warming above 2 °C (Mora et al., 2018). All these three assets class are seen as proper consideration by investors and other stakeholders for diversification and hedging against risk, and our study set out to explore the dynamic linkages of green bonds with renewable energy and the crypto market as set out in our research questions, RQ1 and RQ2.

To achieve the objective, dynamic conditional correlation (DCC), Diebold and Yilmaz (2012) and Baruník and Křehlík (2018) models were applied to multiple time series of daily prices for key market indexes. Namely, the S&P green bond index (RSPGB) as a proxy for green bond; the S&P global clean energy index (RSPCE) and ISE global wind energy (RIGW)as proxies for the renewable energy market, and; Bitcoin (RBIT) and Ethereum (RETHER) as proxies for the crypto market. The daily prices of these constituent series are considered from October 3, 2016, to February 23, 2021. For brevity, we summarize the results as follows. The summation of the coefficients of DCCa and DCCb are less than 1 which ensures stationarity. Second, the application of Diebold and Yilmaz (2012) reveals that green bond (RSPGB) is a net receiver of volatility. The dynamic conditional correlation (DCC) dictates that there is no dynamic linkages of volatility spillover from green bond to energy and crypto market in the short run. In contrast, energy market (RIGW) and cryptocurrency (RETHER) are the largest and least contributors to the volatility transmission, respectively. As green bond net receiver of the volatility, it confirms that it is marginally connected with energy and crypto market. This weak dynamic linkage appears because of lower degree of competition (Naeem et al., 2021a, 2021b). Third, as regards Baruník and Křehlík (2018) model, we notice that the magnitude of the total spillover is higher in the long-term than in the immediate term. In exploring RQ1 and RQ2, this evidence supports the findings of Reboeredo and Ugolini (2020), Pham (2021) but does not corroborate that of Tiwari et al. (2022).

## Implications and limitations of the study

Despite the fact that there is a wealth of information on diversification, there is little information in the literature about cryptocurrencies and their benefits for diversification, particularly with respect to the sustainability aspect. The findings from this paper lend the credence to market participants, investors, portfolio diversification and policymakers. Due to the marginal connectedness or dynamic linkage of green bonds with energy and the crypto market, green bonds help investors hedge the risks generated from aforesaid markets. By analysing the expected returns and risks of cryptocurrencies, alternative investments, and sustainable assets, this study adds to the MPT by demonstrating that expected returns in relation to risks need to be taken into account when investing assets in a portfolio. Additionally, by demonstrating that cryptocurrencies boost an investment portfolio's risk-adjusted returns, this study meets the MPT's requirement that investors assess an investment portfolio's diversification using economically diverse assets and maximise expected return relative to the risk of the portfolio. The information on investment portfolios, cryptocurrencies, and investment diversification is improved by this study. Furthermore, this study has ramifications for financial institutions and investment firms because it highlights the need to take cryptocurrencies into account as diversity assets in their investment portfolios.

In addition, for investors, a wise investment decision will be in holding all these assets class in the short run only as there is no dynamic linkages in the short run, which mitigates investors' risk. Further, it also furnishes an insight into the belief of policymakers as green bonds are considered one of the feasible ways to obtain sustainability goals in an environment that proves worthy market in attracting the attention of investors. On this note, policymakers are encouraged to implement viable strategies to support the green bond market and make it more robust to exogenous shocks. As time passes, the magnitude of the spillover increases, hence, it will be pertinent for the investors to diversify the risk for portfolio composition encompassing green bonds, renewable energy, and cryptocurrency. Moreover, the green bond issuer is also recommended to use this green bond to achieve low-carbon investment goals and avoid financial market turbulence.

The findings obtained from this paper are subject to two major limitations. First, the sample for cryptocurrencies is small as we have included only two series namely, Bitcoin and Ethereum. Second, the present study has not incorporated the volatility connectedness in crisis situations like the current Russia-Ukraine war. Future studies may consider the portfolio weight and hedge ratio amongst green bonds, renewable energy and crypto-market for optimal portfolio diversification. Our advancement of the application of multiple tests also opens a new avenue for further work employing wavelet analysis, counterfactual analysis and quantile regression to examine the connectedness between specific asset classes. In addition, out-of-sample forecasting for volatility can also be considered in future.
